# Supplementary material for: The impact of coronavirus disease 2019 on frozen-thawed embryo transfer outcomes
Source: PeerJ. 2024 Sep 26;12:e18112. doi: 10.7717/peerj.18112 (PMC11439401; doi:10.7717/peerj.18112)
Supplement: Supplemental Information 3 [file peerj-12-18112-s003.pdf]

Name: FET cycle number:

1. Have you been vaccinated against COVID-19 ?

   Yes or No

2. Have you been infected with COVID-19 ?

   Yes or No

(Please continue to fill in if infected)

3. How did you diagnose COVID-19 ?

   Nucleic acid testing

   Antigen testing

   Symptom-based diagnosis (combination with epidemiological history)

4. What were your symptoms when you were infected with COVID-19?

   asymptomatic

   fever, cough, phlegm, sore throat, runny nose, headache, dizziness, body aches, chest tightness, weakness, shortness of breath

   nausea, vomiting, abdominal pain, diarrhea, constipation

   loss of smell and taste, loss of memory, irritability, anxiety, insomnia

5. How long did your symptoms disappear when you were infected with COVID-19?

   1-3 days; 4-7 days; 2 weeks; 3 weeks; 4 weeks; more than 4 weeks

6. How long was it between your infection and the embryo transfer?

   within 1 month;1~ 2 months; 2~3 months; more than 3 months

7. Have your periods changed since COVID-19 ?

   no change;

   prolonged menstrual cycle; shortened menstrual cycles;

   decreased menstrual volume; increased menstrual volume;

   prolonged menstruation; shorter menstrual periods
